# Multifaceted Targeting of the Chromatin Mediates Gonadotropin-Releasing Hormone Effects on Gene Expression in the Gonadotrope

**DOI:** 10.3389/fendo.2018.00058

**Published:** 2018-02-27

**Authors:** Philippa Melamed, Majd Haj, Yahav Yosefzon, Sergei Rudnizky, Andrea Wijeweera, Lilach Pnueli, Ariel Kaplan

**Affiliations:** ^1^Faculty of Biology, Technion—Israel Institute of Technology, Haifa, Israel

**Keywords:** gonadotropin-releasing hormone, gonadotrope, luteinizing hormone, follicle-stimulating hormone, chromatin, histone, transcription, gene

## Abstract

Gonadotropin-releasing hormone (GnRH) stimulates the expression of multiple genes in the pituitary gonadotropes, most notably to induce synthesis of the gonadotropins, luteinizing hormone (LH), and follicle-stimulating hormone (FSH), but also to ensure the appropriate functioning of these cells at the center of the mammalian reproductive endocrine axis. Aside from the activation of gene-specific transcription factors, GnRH stimulates through its membrane-bound receptor, alterations in the chromatin that facilitate transcription of its target genes. These include changes in the histone and DNA modifications, nucleosome positioning, and chromatin packaging at the regulatory regions of each gene. The requirements for each of these events vary according to the DNA sequence which determines the basal chromatin packaging at the regulatory regions. Despite considerable progress in this field in recent years, we are only beginning to understand some of the complexities involved in the role and regulation of this chromatin structure, including new modifications, extensive cross talk, histone variants, and the actions of distal enhancers and non-coding RNAs. This short review aims to integrate the latest findings on GnRH-induced alterations in the chromatin of its target genes, which indicate multiple and diverse actions. Understanding these processes is illuminating not only in the context of the activation of these hormones during the reproductive life span but may also reveal how aberrant epigenetic regulation of these genes leads to sub-fertility.

## Introduction

**Figure 1:**
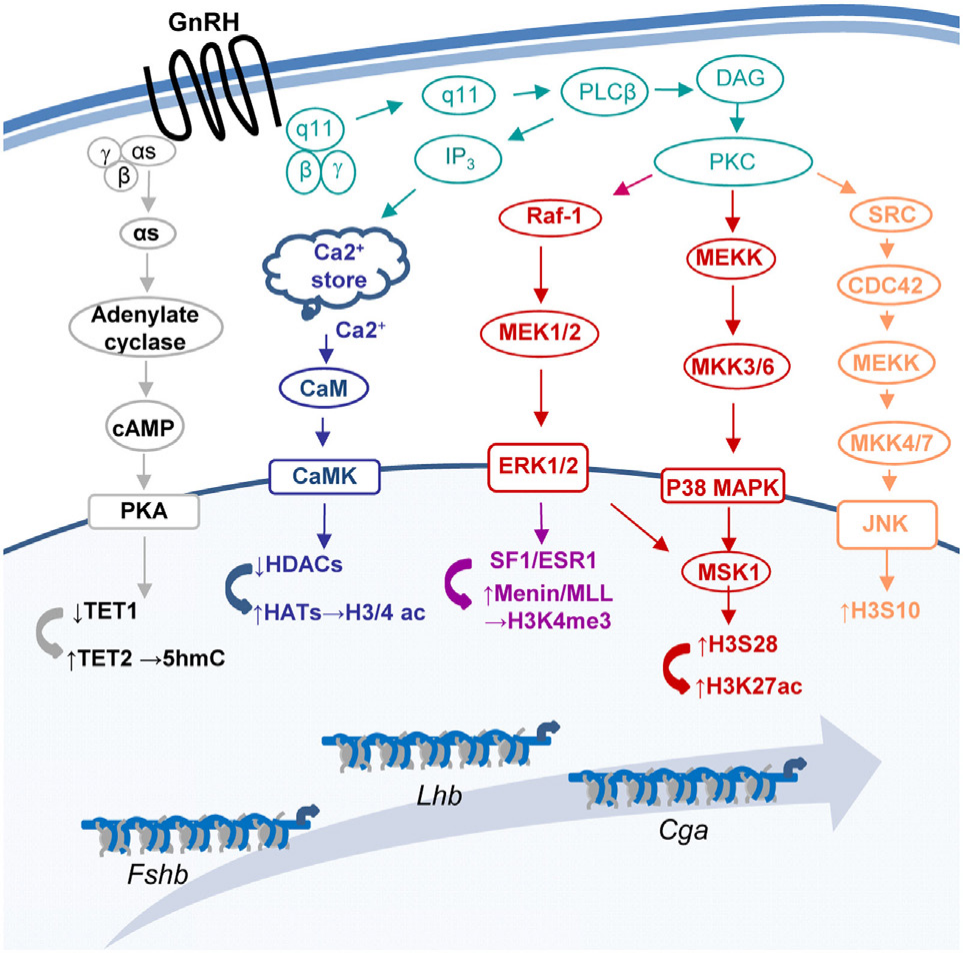
Some of the pathways through which gonadotropin-releasing hormone (GnRH) modifies the chromatin at the three gonadotropin subunit genes. GnRH binds its receptor (GnRHR) to activate a number of pathways that modify the chromatin and lead to changes in expression of the genes encoding the common gonadotropin α-subunit (*Cga*) and the hormone specific β-subunits of luteinizing hormone (*Lhb*) and follicle-stimulating hormone (*Fshb*).

Gonadotropin-releasing hormone (GnRH) regulates the expression of multiple gonadotropic genes [e.g., Ref. (1–4)], to control population size (5–7), differentiation (8), morphology, and migration (9–11) as well as response to other regulatory hormones [e.g., Ref. (12–15)]. The GnRH receptor (GnRHR)-induced activation of MAP- and other kinase pathways (16–18), culminates in expression and/or activation of gene-specific transcription factors [e.g., Ref. (19–25)], allowing them to bind the DNA and stimulate transcription, often *via* recruitment of coactivators which catalyze chromatin modifications [e.g., (19, 26, 27)]. However, some MAPKs are associated with the chromatin, where they phosphorylate histones (28, 29), and GnRH also targets several chromatin and DNA-modifying genes directly [Ref. (8, 19, 30, 31); Figure 1], indicating much broader mechanisms for moderating chromatin organization.

Although the nucleosome is usually highly stable, chromatin structure is dynamic, and this plays a role in determining the accessibility of regulatory DNA, *via* chromatin modifications which alter nucleosome behavior (32). Many histone modifications occur on the N-terminal tails; some affect contact with DNA through altering histone charge, while others “write” a signal which is recognized by protein effectors [“readers” (33, 34)]. However, chromatin-modifying complexes often comprise multiple components with activities to both “read” and “write” various modifications including those on DNA, as well as ATP-dependent remodeling enzymes that reposition or reorganize the nucleosomes to facilitate transcription initiation and also transition of RNAPII through nucleosomes. Such a diversity of distinct enzymes in a single complex allows for sophisticated dialog and cross talk (33, 35, 36).

This short review will highlight the multiple ways through which GnRH targets the chromatin at the gonadotropin genes which, in addition to clarifying the regulation of these genes during development, should lead to greater understanding of how aberrant epigenetic regulation of these genes might underlie fertility problems.

## Histone Acetylation and Deacetylation

By neutralizing the positive charge of lysines on histone N-terminal tails, acetylation at this residue disrupts histone–DNA interactions to make chromatin more accessible, and is thus commonly found at active regions of the genome. Accordingly, basal expression levels of the three gonadotropin genes in partially differentiated gonadotrope-precursor αT3-1 cells closely correlate with levels of H3 acetylation and inversely with H3 occupancy (31). Although H3 and H4 undergo acetylation at various lysines, their differential acetylation at the N-terminus may have a redundant role in transcription such that, in certain contexts, the cumulative charge neutralization influences the transcriptional outcome of a gene more than the acetylation of any specific lysine (37, 38). However, acetylated lysines can be recognized by bromodomain proteins, including multiple chromatin-modifying and remodeling enzymes, such that this modification may well function as a specific recognition site for additional transcriptional activators (39). GnRH increases gonadotrope H3 acetylation, seen both globally and at the 5′ end of the *Cga* gene which encodes the gonadotropin common α subunit, indicating that this comprises part of the regulatory mechanism of GnRH-induced upregulation of gene expression (31). Histone acetylation is catalyzed by the histone acetyl transferase (HAT) activity of several common transcriptional coactivators, some of which have been shown to mediate hormonally-induced expression of the gonadotropin genes (19, 26, 40).

The opposing activity is executed by histone deacetylases (HDACs) which repress expression of the gonadotropin β-subunit genes in gonadotrope precursor cells (22, 41). Exposure of these cells to GnRH allows de-repression of the *Lhb* and *Fshb* genes as a result of activation of calmodulin-dependent kinases, which phosphorylate class II HDACs associated with the gene promoters, leading to their nuclear export (17, 22, 41). A similar mechanism may be responsible for the repression of *Fshb* in the more fully differentiated LβT2 cell line, as GnRH or an HDAC inhibitor facilitated its expression quite specifically, indicating repression by HDACs, which is overcome by GnRH (42, 43).

Both HAT and HDAC enzymes are characteristically found in large multiprotein complexes whose recruitment may follow other chromatin modifications, while they often also recruit additional modifying enzymes to these *loci* to provide elaborate cross talk (44). In partially differentiated gonadotropes, SIN3A and SMRT corepressors were found at the *Fshb* gene promoter, together with class I and class II HDACs. GnRH treatment caused loss of SIN3A and HDAC association, and various components were displaced following knockdown of HDAC4 or either of the co-repressors, suggesting their central roles in this complex (22). The nature of the repressive HDAC complex at the *Lhb* gene is less clear, but as this gene is regulated by DNA methylation and by TET1 (8), additional modifying enzymes are clearly involved: HDAC-containing complexes are often recruited by methylated DNA-binding proteins (MBPs) or DNA methyl transferases (DNMTs), and the HDAC-containing PRC2 complex contains also EZH2 which represses transcription by catalyzing H3K27me3 (35, 45).

On the other hand, HATs are often associated with other chromatin-activating enzymes, including additional HATs and chromatin-remodeling enzymes, as well as chromodomain proteins that bind H3K4me3. The chromodomain-helicase–DNA-binding domain protein 1 (CHD1) in yeast SAGA/SLIK HAT complexes is recruited to gene promoters in this way, thereby facilitating HAT activity (46) while also altering nucleosomal stability or turnover. Notably, CHD1 is found at the active *Cga* promoter, but its levels were significantly reduced after disruption of enhancer function, and it was not found at the *Lhb* gene promoter (47, 48). This drop in CHD1 association with the *Cga* gene was accompanied by a drop in histone acetylation and H3K4me3 levels which suggests a common pathway/complex of recruiting these enzymes, although the exact mechanism has still to be elucidated. We did not measure histone phosphorylation in this context, but histone acetylation has also been linked to phosphorylation, since discovery that the HAT GCN5 binds preferentially phosphorylated H3S10 which couples these modifications in EGF-induced transcription (49–51).

## Histone Phosphorylation

Given that GnRHR signaling involves activation of several MAPKs, it is not surprising that GnRH induces histone phosphorylation, seen globally and at the gonadotropin promoters (31). Some of the MAPKs activated by GnRH, including JNK, can target histone phosphorylation directly (28, 29) and this kinase is responsible for H3S10p at the *Cga* promoter. The phosphorylation of H3S28 at the *Cga* 5′end is *via* GnRH-activation of MSK1, which is a downstream target of ERK and p38 MAPK (31).

By introducing a positive charge, this phosphorylation would be expected to destabilize the DNA–histone interactions and thus aide in passage through the nucleosome (32). However, the role of H3 phosphorylation in transcriptional regulation is still not fully understood, as it is seen at both repressed and active genes (52, 53). Notably, phosphorylation of H3S10 on the *Cga* gene 5′ end/promoter, even though it is increased in response to GnRH, appears to have little function on on-going and/or hormonally upregulated *Cga* transcription, as no effect was noted when its basal levels were reduced by over 90%. However, H3S28p at the first nucleosome in the transcribed region appears to play a role in elongation, presumably facilitating RNAPII transition through this nucleosome, which is likely aided by GnRH-induced H3K27ac (31).

H3S10 or S28 phosphorylation may also alter the affinity of additional chromatin binding proteins to their targets, as reported above for GCN5 (49, 54). At the *Cga* gene, H3K9ac appeared independent of H3S10p, while inhibition of H3S28p was accompanied by a drop in global levels of H3K27ac, although H3S18ac was unaffected (31). In other contexts, H3S10p plays a more crucial role in the activation of repressed genes, as it can trigger the displacement of the HP1γ repressor, and facilitate recruitment of the SWI/SNF chromatin-remodeling enzyme Brg1 and RNAPII (55, 56). The interpretation of histone phosphorylation thus appears to be highly context specific, involving cross talk with neighboring histone residues to determine the precise outcome.

## Histone Methylation

The mono-, di- or trimethylation of specific lysines by histone methyltransferases and demethylases distinguishes transcriptionally active from inactive chromatin domains [reviewed by Ref. (57)]. At the gonadotropin genes, levels of H3K4 trimethylation (H3K4me3) which marks the 5′ ends and/or promoters of all actively transcribed genes and is essential for transcription initiation (58), correlate well with basal expression levels and increase following GnRH exposure (27). Mammals have six distinct complexes capable of catalyzing this modification, with multiple subunits affording different mechanisms of recruitment and regulation (59, 60). The mixed-lineage leukemia (MLL)-COMPASS-like complex 1/2 is recruited to the gonadotropin genes during their upregulation by GnRH and is responsible for the GnRH-induced increase in H3K4me3 at all three gonadotropin promoters (27). Unique among the Set1/COMPASS-like complexes, the MLL1/2 complex contains menin (60), which interacts with various gene-specific transcription factors including ERα (61, 62) and Sf-1 (27). The GnRH-induced association of menin with the β-subunit genes, is dependent on Sf-1. Sf-1 recruits ERα to the *Lhb* promoter, after GnRH-induced modification of both factors. Both of these factors appear to play roles in the recruitment of the MLL1/2 complex and thus also H3K4me3 at these genes (12, 21, 27).

The GnRH-induction of H3K4me3 at the gonadotropin genes alters nucleosomal occupancy, and there was increased association of H3 at the promoters following inhibition of menin which was not overcome by exposure to GnRH (27). H3K4me3 was previously reported to play a role in maintaining low nucleosomal occupancy, likely due to its ability to bind chromatin-remodeling enzymes such as CHD1 and ISWI (46, 63, 64). As described above, it also helps recruit HAT complexes (65, 66) and TFIID (67). However, whether the menin-dependent loss of H3 at the gonadotropin gene promoters following GnRH exposure involves such a mechanism is not yet clear.

Trimethylation of H3K36 (H3K36me3) in the coding regions of all three gonadotropin genes also increases following exposure to GnRH (27). This is likely a direct consequence of increased transcription rates as it is catalyzed by Set2 which is recruited by the elongating S2p form of RNAPII [reviewed by Ref. (68)], and the increased level of this modification at the gonadotropin genes correlates with elevated association of RNAPII (27). H3K36me3 is reported to have a number of roles including regulating histone exchange (69), suppression of initiation through recruitment of DNMT3b and intragenic DNA methylation (70), RNA processing (71), chromatin organization (72), and others, which have yet to be explored in this context.

## H2B Ubiquitination

Monoubiquitinated H2B at lysine 120 (H2BK120ub) is generally associated with actively transcribed genes, as first reported in yeast, although its precise function in mammals has been controversial. It is reportedly required for recruitment of the Set1/COMPASS complex to gene promoters due to its recognition by one of the complex subunits. This ubiquitin is later removed by a component of the SAGA HAT complex to allow recruitment of the kinase that phosphorylates RNAPII at S2, thus signaling promoter escape and elongation (68, 73). However, it appears that for many mammalian genes H2BK120ub is found primarily in the transcribed region where it plays a role in the reassembly of nucleosomes in the wake of elongating RNAPII (74–76). The presence and requirement of H2BK120ub at mammalian gene promoters has been disputed, perhaps in part due to the different chromatin organization in the various genes studied and the associated diverse transcription dynamics (see below), and possibly also to earlier experimental protocols which mapped its exact genomic location with poorer resolution.

The apparent lack of requirement for H2BK120ub at some mammalian promoters is likely due to the more numerous complexes that can catalyze H3K4me3 than found in yeast. As described above, these complexes contain distinct subunits which allow recruitment of the lysine methyl transferase (KMT) complex through diverse proteins including transcription factors. Accordingly, there is no apparent correlation between levels of promoter H2BK120ub and expression levels of the three gonadotropin genes, nor with levels of H3K4me3 at their promoters. However, GnRH induces a major increase in H2BK120ub levels globally, and specifically in the coding regions of the gonadotropin genes, in keeping with the changes in coding region H3K36me3 described above (27). The fact that this elevation in H2BK120ub was also noted globally suggests that it is a common event at the multiple genes upregulated by GnRH.

## Histone Citrullination

Histone citrullination, in which histone tail arginine residues are converted by peptidylarginine deiminase (PAD) enzymes to citrulline has been observed but is still poorly understood. The citrullination is thought to induce chromatin decondensation, and was recently shown to be particularly crucial for transcriptional activation during early embryonic development (77, 78). Notably, PAD family members are highly expressed in female reproductive tissues, have been indicated to play a critical role in female reproduction, and a correlation was seen particularly between PAD2 expression levels, and stages of the estrous cycle (79). Moreover, GnRH was reported to induce PAD2 nuclear localization in gonadotropes, where it stimulates citrullination of H3 at R2, R8, and R17. The inhibition of this activity was seen to blunt the GnRH stimulatory effect on the gonadotropin β-subunit genes, suggesting that histone citrullination mediates part of the GnRH effect, although it has yet to be shown whether it targets the gonadotropin genes directly and the exact mechanism involved (30).

## The DNA-Modifying TET Enzymes

DNA methylation is generally considered a stable mechanism to repress gene expression, often in concert with repressive histone modifications. However, since the discovery of the TET family of enzymes (80, 81), it has become clear that methylated cytosines (5mC) can be hydroxylated to 5hmC, often imparting a very different function as the 5hmC modified-DNA is not recognized similarly by some of the 5mC-binding proteins (82) which can thus lead to de-repression through “functional demethylation.” The maintenance DNMT, DNMT1 also binds 5hmC DNA with much lower affinity than to 5mC DNA, leading to a passive demethylation in replicating cells (83, 84). More recently, the TET enzymes were noted to catalyze additional modification of 5hmC, to bases that are quickly removed by the base-excision repair mechanism, in a form of active demethylation (85, 86). Despite this potential of the TET enzymes to relieve inhibition of gene expression, TET1 is also sometimes found in repressor complexes in association with other inhibitory chromatin-modifying enzymes (87–89).

In this context, TET1, which is highly expressed in gonadotrope-precursor cells, was found to repress the *Lhb* gene in association with promoter H3K27 methylation, possibly playing a role in recruitment of the KMT enzyme to this locus. *Tet1* expression is downregulated to allow the precursor gonadotropes to complete their differentiation, following exposure to GnRH and also in response to estrogens or androgens *via* steroid receptors that bind the *Tet1* promoter (8). With developmental or experimentally-induced downregulation of TET1, it is replaced at the *Lhb* gene promoter by TET2 which hydroxymethylates the methylated CpGs on this gene promoter so, in concert with the GnRH-activated transcription factors, facilitating *Lhb* expression (8). In this way, the exposure of the partially differentiated gonadotrope precursors to GnRH promotes their final differentiation via downregulation of *Tet1* and the ensuing elevation in gonadal steroids provides the feedback to keep *Tet1* repressed.

## Nucleosomal Organization and Remodeling

**Figure 2:**
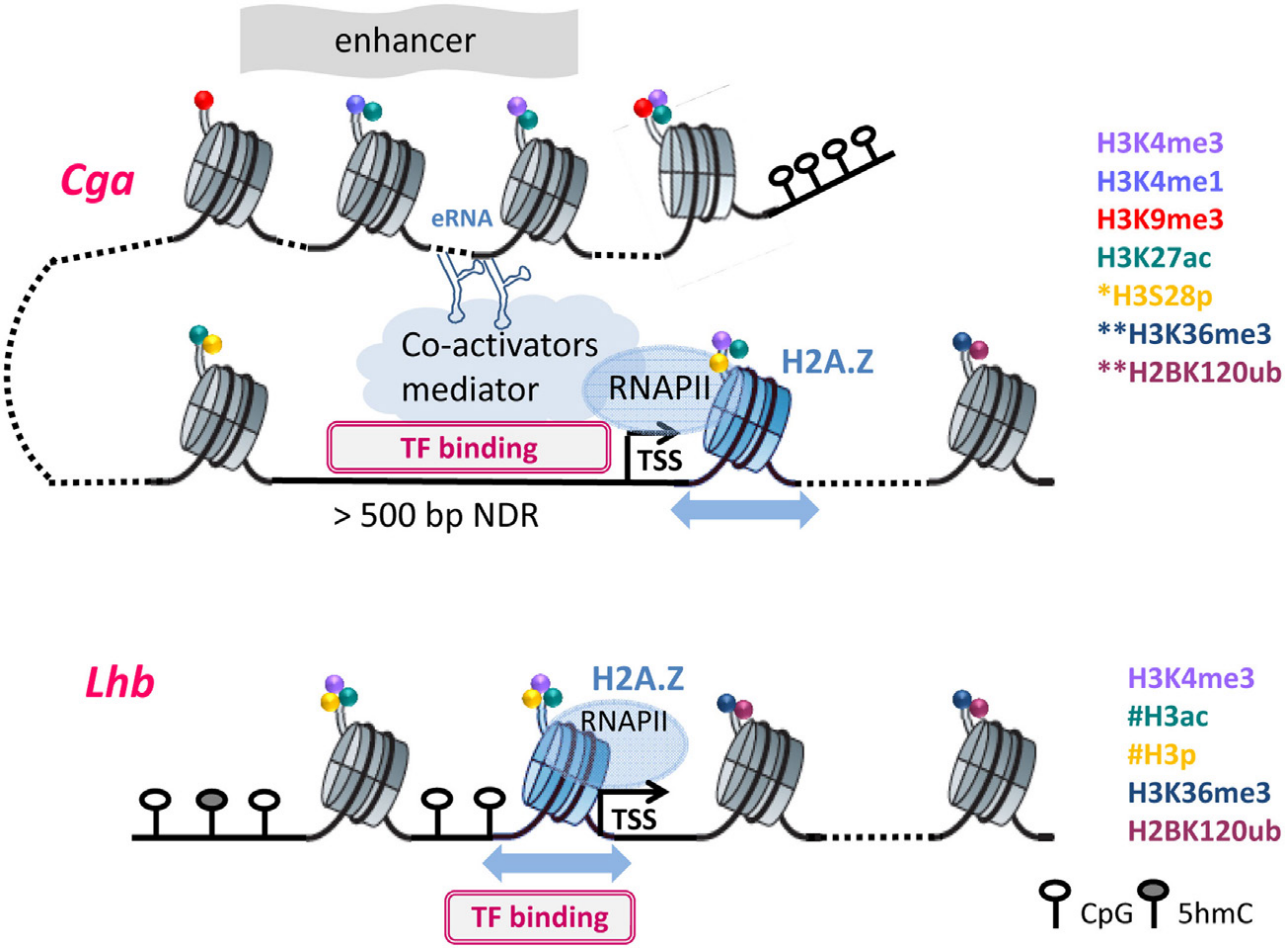
The different chromatin organization necessitates distinct modifications to facilitate transcription: Chromatin organization at *Cga* and *Lhb* regulatory regions are shown in their active states, with DNA and histone modifications as noted. *S28p was examined only at the proximal region of *Cga*; **at the downstream nucleosomes, only H3K36me3 and H2K120Bub were assessed; ^#^histone acetylation and phosphorylation are inferred but were not measured directly on the *Lhb* promoter. Furthermore, the nucleosomal density on the *Lhb* proximal promoter precluded precise mapping of H3K4me3. Dotted lines represent DNA packaged into additional nucleosomes that are not shown, and the blue arrows represent nucleosome mobility due to H2A.Z incorporation. Further details can be found in the text.

For all three gonadotropin genes, nucleosome levels drop following GnRH treatment, reflecting histone displacement (27, 31). However, the organization of the chromatin at the *Cga* and *Lhb* gene promoters in functional gonadotropes differs markedly, in accordance with their distinct expression levels and means of regulation. In the gonadotropes, the *Cga* proximal promoter, similar to that of many other highly expressed genes but unlike its state in non-gonadotropes, is exposed and accessible to transcription factors, such that transcription may be initiated quite easily (48). Upregulation of *Cga* expression by GnRH is, therefore, likely directed primarily at the level of RNAPII promoter escape and elongation such that after exposure to GnRH it is quickly released from the promoter (27, 48). This is reflected in much higher levels of RNAPII at the *Cga* than *Lhb* promoter in unstimulated mature gonadotropes, and the fact that the first nucleosome in the coding region, characteristic of genes with high RNAPII occupancy (90), is positioned 10 bp further downstream than at the *Lhb* gene. RNAPII transition through this nucleosome is clearly facilitated by the incorporation of a histone H2A variant which allows greater nucleosomal mobility (48), and likely further enhanced by GnRH-induced modifications targeting the histones in this nucleosome (Figure 2).

This organization of the *Cga* gene differs fundamentally from that of *Lhb* whose proximal promoter is packaged into a nucleosome (Figure 2) that encompasses binding sites of Sf-1, Pitx-1, and Egr-1, which activate this gene (91, 92). The initiation of transcription must thus require reorganization to allow access of these factors to the DNA, likely facilitated by the incorporation of H2A.Z at the *Lhb* promoter (48, 93). The binding of TFs to sites that are buried inside the nucleosome is thought to be modulated by the thermally driven spontaneous “breathing” (94). This involves partial wrapping and unwrapping of the DNA at the entry and exit to nucleosome which can expose the binding site, such that its ability to bind is also a function of the distance of the binding site from the nucleosome dyad (95, 96). These fluctuations, which are typically much faster than rates of nucleosome repositioning, likely work together with increased nucleosome mobility to facilitate the initial access of Sf-1 and/or Pitx-1 to their binding sites (93). Binding of either of these “pioneer” factors would destabilize the nucleosome both through the binding itself and the recruitment of the histone-modifying enzymes described above, as well as quite possibly ATP-dependent chromatin-remodeling enzymes. Clearly there is much more work to be done in order to understand the various components and their intricate roles in the activation of this gene, and also whether the nucleosomes at the *Fshb* gene promoter are similarly organized and modified following GnRH exposure.

## Concluding Comments

The organization of the chromatin at the gonadotropin genes and its GnRH-induced modifications that facilitate transcription is crucial in understanding how these genes are activated during the reproductive lifespan, but may also have implications in non-pituitary GnRHR-expressing cancer cells. There is increasing indication, however, that much larger distal genomic regions function to determine expression of specific genes, as shown for the eRNA that regulates *Cga* chromatin (47, 97). The likelihood that genes are regulated by a variety of distal enhancers in various scenarios (97), points to highly complex gene-regulation in distinct developmental, hormonally activated and pathological contexts, while emphasizing the importance of the chromatin architecture in extensive genomic regions. As the epigenome is susceptible to external perturbations, elucidation of the full complement of elements that regulate gonadotropin gene expression and their chromatin organization will further our understanding of abnormal gonadotropin levels and the ensuing pituitary-origin reproductive disorders, while opening the way for epigenetic targeting as a basis for fertility drug development and treatment.

## Author Contributions

All authors contributed to this review, and all have read and approved the final manuscript.

## Conflict of Interest Statement

The authors declare that the research was conducted in the absence of any commercial or financial relationships that could be construed as a potential conflict of interest. The handling editor declared a past coauthorship with one of the authors PM.
